# Update on Novel Therapeutics for Primary CNS Lymphoma

**DOI:** 10.3390/cancers13215372

**Published:** 2021-10-26

**Authors:** Lauren R. Schaff, Christian Grommes

**Affiliations:** Memorial Sloan Kettering Cancer Center, Department of Neurology, Weill Cornell Medicine, New York, NY 10065, USA; GrommesC@mskcc.org

**Keywords:** PCNSL, CNS lymphoma, methotrexate, novel therapies, novel therapeutics

## Abstract

**Simple Summary:**

Primary central nervous system lymphoma is a rare and aggressive form of non-Hodgkin lymphoma. While it is highly responsive to first-line chemo and radiation treatments, rates of relapse are high, demonstrating the need for improved therapeutic strategies. Recent advancements in the understanding of the pathophysiology of this disease have led to the identification of new potential treatment targets and the development of novel agents. This review aims to discuss different targeted strategies and review some of the data supporting these approaches, and discusses recently completed and ongoing clinical trials using these novel agents.

**Abstract:**

Primary central nervous system lymphoma (PCNSL) is a rare lymphoma isolated to the central nervous system or vitreoretinal space. Standard treatment consists of cytotoxic methotrexate-based chemotherapy, with or without radiation. Despite high rates of response, relapse is common, highlighting the need for novel therapeutic approaches. Recent advances in the understanding of PCNSL have elucidated mechanisms of pathogenesis and resistance including activation of the B-cell receptor and mammalian target of rapamycin pathways. Novel treatment strategies such as the Bruton’s tyrosine kinase (BTK) inhibitor ibrutinib, phosphatidylinositol-3 kinase (PI3K) inhibitors, and immunomodulatory drugs are promising. Increasingly, evidence suggests immune evasion plays a role in PCNSL pathogenesis and several immunotherapeutic strategies including checkpoint inhibition and targeted chimeric antigen receptor T (CAR-T) cells are under investigation. This review provides a discussion on the challenges in development of targeted therapeutic strategies, an update on recent treatment advances, and offers a look toward ongoing clinical studies.

## 1. Introduction

Primary central nervous system lymphoma (PCNSL) is a rare variant of extra-nodal non-Hodgkin lymphoma that affects only the central nervous system (CNS) and/or vitreoretinal space in the absence of systemic involvement. This differs from secondary CNS lymphoma (SCNSL) in which CNS disease may represent progression or a relapse of a systemic lymphoma that may harbor different genetic features. CNS lymphoma affects approximately 1600 people per year in the United States and is more common in the elderly, with a median age of 67 at diagnosis [1]. Immunodeficiency is a risk factor for PCNSL, but the disease may also occur sporadically in immunocompetent patients. This review will focus on advances in the treatment of immunocompetent patients with PCNSL.

The presentation of PCNSL may be varied and diagnosis requires a high degree of clinical suspicion. Symptoms may be focal, related to direct tumor involvement of the eye, brain, or spinal cord, or may be non-specific. Up to 50% of the time, patients present with cognitive decline and behavioral changes that may not prompt immediate neuro-imaging [2]. When imaging is obtained, magnetic resonance imaging (MRI) with and without contrast is the modality of choice. PCNSL often presents with characteristic homogeneously enhancing, diffusion restricting, deep brain lesions. Full disease staging requires an MRI of the spine, a lumbar puncture, and a slit lamp examination. To differentiate a PCNSL from SCNSL, systemic work up is required. A positron emission tomography (PET) scan of the body should be performed. If a PET cannot be obtained, patients should undergo computed tomography (CT) of the chest/abdomen/pelvis to look for lymphadenopathy, paired with a bone marrow biopsy and a testicular ultrasound in men.

PCNSL is highly chemo- and radio-responsive. While surgical sampling is often required for diagnosis, tissue studies suggest involvement of the whole brain [3]. Multiple retrospective studies have failed to demonstrate a survival benefit with extensive surgery [2,4,5] and as a result, resection is typically not pursued.

Chemotherapy alone, particularly methotrexate (MTX)-based treatment, results in dramatic clinical and radiographic responses, often inducing remission. While MTX is broadly considered an important component of first-line treatment, there is a lack of consensus regarding the optimal chemotherapy regimen. Polychemotherapy regimens that include MTX are associated with improved response rates and progression-free survival (PFS) as compared to MTX monotherapy [6]. However, there is a paucity of prospective randomized data comparing MTX-based regimens and as a result, different practice approaches have developed. Common regimens include rituximab/MTX/procarbazine/vincristine (R-MPV) [7], MTX/temozolomide/rituximab) (MT-R) [8], MTX/cytarabine/thiotepa/rituximab (MATRix) [9], rituximab/MTX/carmustine/teniposide/prednisolone (R-MBVP) [10], and rituximab/MTX (R-M) [11]. The optimal dose of MTX is not known, though most practitioners agree that a dose of at least 3 g/m^2^ is required for adequate penetration of the CNS [12]. Some regimens utilize dosages up to 8 g/m^2^ [8] though toxicity often necessitates dose reductions and there is no clear benefit to these higher doses. Ultimately, choice of regimen often comes down to institutional and practitioner preference.

Without a consolidation strategy to follow MTX-based chemotherapy, the likelihood of PCNSL relapse is high, with a median PFS of 21.5 months after a complete response (CR) [13]. Historically, consolidation consisted of whole brain radiation therapy (WBRT) though it is unclear whether WBRT results in an overall survival (OS) benefit and it is associated with long-term neurotoxicity [13]. Whether a lower than standard dose of WBRT adequately addresses the issue of neurotoxicity remains to be seen [14]. Increasingly, myeloablative high-dose chemotherapy followed by autologous stem cell transplant (HDC-ASCT) is the preferred consolidation strategy for eligible patients. Such an approach after MTX-based therapy yields response rates of more than 90% [15] with median PFS of 74 months in one study [15] and not-reached in others [16,17]. For patients who are elderly or frail, non-myeloablative chemotherapy with high-dose cytarabine with or without etoposide may be considered [8,10,18]. Maintenance chemotherapy in lieu of consolidation is also a reasonable treatment approach [19,20]. In clinical trials, targeted or immunotherapies are also being explored for this purpose.

Despite aggressive treatment for PCNSL, approximately 15% of patients have refractory disease [21] and relapse rates are high, particularly in patents who are not candidates for HDC-ASCT. Traditional strategies for salvage therapy include MTX-rechallenge [22,23], alternate cytotoxic chemotherapy regimens [24,25,26], and WBRT [27,28]. Prognosis for relapsed disease is poor with a PFS of only about a year with aggressive salvage therapy [29]. As a result, there is a desperate need for novel therapeutic strategies. Recent developments in the understanding of the pathogenesis of PCNSL have led to the investigation and use of new, targeted approaches.

## 2. Pathophysiology

A vast majority of PCNSL cases are comprised of a diffuse large B cell lymphoma (DLBCL) and express pan-B cell markers CD20, CD19, CD22, and CD79a. Other lymphomatous malignancies such as T-cell lymphoma, Burkitt lymphoma, and lower grade lymphoproliferative neoplasms have been described but are less common and may warrant special considerations with regard to treatment strategy.

Histologically, DLBCL in the brain is highly proliferative with an angiocentric growth pattern. Based on the Hans criteria [30] and immunohistochemistry, a majority (>75%) of PCNSL cases are classified as activated B-cell-like (ABC)/nongerminal center subtype [31,32,33]. However further evidence with immunoglobulin heavy chain gene mutational signatures and immunophenotyping suggest PCNSL has germinal center origin or exposure [31,34,35,36,37] and increasingly, there is evidence PCNSL may demonstrate an overlapping state of differentiation with concurrent expression of germinal center markers such as BCL6 and activation markers such as cyclin D2 or MUM1/Interferon Regulatory Factor 4 (IRF4) [31,38]. Ultimately the relevance of differentiating between ABC or germinal center subtype in PCNSL is unclear and unlike in systemic lymphoma where the ABC subtype confers a poorer prognosis, there is no clear survival advantage associated with any particular subtype of PCNSL [39].

Single nucleotide variants and copy number alterations are frequent genetic events in PCNSL. *MYD88, CD79B*, *CARD11*, and *TNFAIP3* are amongst the most frequently mutated genes. Systemically, *MYD88* mutations are associated with the ABC subtype; but in PCNSL, *MYD88*, and *CD79B* have been described in both ABC and GCB subtypes of disease. *MYD88* missense mutations (most common L265P [40]) lead to constitutive activation of the TLR pathway [41], while alterations in *CD79B* activate the BCR pathway [42]. Mutations in the coiled-coil domain of *CARD11* result in downstream activation of both pathways [43] while alterations to *TNFAIP3* can result in a loss of pathway inhibition. Ultimately, the BCR/TLR pathways result in upregulation of nuclear factor kappaB (NFκB), a protein transcription factor that promotes neoplastic proliferation and prevents apoptosis [44]. Copy number alterations may also contribute to pathogenesis. Losses are common at 6p21.33 (*HLA-B*, *HLA-C*), 6q21-23 (*TNFAIP3*), and 9p21.3 (*CDKN2A*). Copy number gains may be seen at 12q (*MDM2*, *CDK4*) and 9p24.1 (*PD-L1*, *PD-L2*). Somatic hypermutation (SHM) is also thought to play a role in PCNSL pathogenesis and may offer further rationale for use of immunotherapy. Genetic features of vitreoretinal lymphoma (VRL) have significant overlap with PCNSL, and result in probable activation of the TLR pathway. Mutations in *MYD88* may be more common in VRL (and not limited to L265P) while *CD79B* mutations appear less common [45]. SHM genes may be similarly mutated.

Increasingly, evidence suggests the tumor microenvironment also plays an important role in PCNSL. IL-10 is a cytokine that may serve as a prognostic biomarker and also appears to lead to activation of signal transducer and activator of transcription 3 (STAT3) [46]. The Janus kinase 2 (JAK2)/STAT3 pathway results in transcription of target genes involved in cellular proliferation, survival, and angiogenesis. STAT3 is expressed in a variety of malignancies including PCNSL [46]. Tumor-associated macrophages (TAMs) interact with PCNSL cells and promote tumor invasion, proliferation, and an immunosuppressed environment. Quantification of TAMs may be important in prognosis [47]. TAMs also overexpress *PD-L1*, suggesting a potential target for immunotherapy.

Overall, PCNSL appears to be biologically distinct from systemic lymphoma and is increasingly considered a separate entity [48,49,50]. Genetic alterations seen in PCNSL including activation of the B-cell receptor (BCR) and Toll-like receptor (TLR) signaling pathways most closely resemble those observed in testicular lymphoma [51,52,53], suggesting similar pathogenesis between these two immunoprivileged sites. Improved understanding of the unique molecular profile of PCNSL has allowed for the recent investigation of multiple targeted strategies (Table 1).

## 3. Molecular Targets

### 3.1. BCR/TLR Pathway

Discovery of alterations involving the BCR and TLR pathways has led to the most significant recent breakthroughs in the treatment of PCNSL. The BCR signaling pathway can be targeted at different signaling nodes. Upstream, the pathway may be downregulated through targeting phosphatidylinositol-3 kinase (PI3K). Downstream, immunomodulatory drugs like lenalidomide may be used to inhibit IRF4, which affects NFκB function. Proteosome inhibitors may prevent release of NFκB to the nucleus, where it results in alteration of gene expression. Unfortunately, proteasome inhibitors are often too bulky to cross the blood–brain barrier (BBB).

Bruton’s tyrosine kinase (BTK), the central signaling node of the pathway, can be targeted with ibrutinib. A prospective study of ibrutinib 560 mg daily in 52 patients with relapsed/refractory PCNSL demonstrated a response rate of 52% [60]. A higher dose of 840 mg daily may result in increased cerebrospinal fluid (CSF) concentration and remains well tolerated [55,59] though the clinical benefit of this higher dosing schedule is unknown and additional data suggests the enzymatic IC_50_ is not proportional to dose [40]. Response to ibrutinib occurs quickly with one ‘window study’ demonstrating a response rate of 83% to only two weeks of single-agent ibrutinib, prior to the addition of further chemotherapy [40]. Notably, these high response rates are in contrast the experience in systemic lymphoma where single agent ibrutinib may result in a response rate of only 25% [62]. While this may be in part due a higher incidence of BCR/TLR alterations in PCNSL such as *MYD88*, it is important to note that even PCNSL patients without obvious genomic alterations in the BCR pathway demonstrate ibrutinib response [60]. It is also worth noting that while concurrent *CD79B* and *MYD88* mutations appear to sensitize systemic lymphoma to ibrutinib [62], this same combination was associated with a poorer response in CNS disease, perhaps due to decreased dependence on the BCR pathway [55]. These mutations appear to coincide in approximately 37% of cases of PCNSL [40]. *CARD11* and *TNFAIP3* mutations are potential sources of ibrutinib resistance given their activity downstream BTK. While this has been described in systemic lymphoma [62,63] and PCNSL with ibrutinib monotherapy [55], adequate responses were seen in patients with these potential resistance mechanisms when ibrutinib was used in combination with cytotoxic chemotherapy [59].

Despite high rates of radiographic response, the progression-free survival provided by ibrutinib monotherapy is less than 5 months, suggesting early development of resistance [55,60]. With ibrutinib combination treatment, that PFS is extended to approximately 9 months in pre-treated patients [59]. Multiple studies are now incorporating ibrutinib into combination therapy, paired with agents such as lenalidomide (NCT03703167), copanlisib (NCT03581942), checkpoint inhibition (NCT04421560, NCT03770416), and traditional chemotherapy (NCT04066920, NCT02315326).

Ibrutinib has been incorporated to the National Comprehensive Cancer Network (NCCN) guidelines for treatment of relapsed/refractory PCNSL. Studies investigating ibrutinib for use in newly diagnosed patients are currently underway (Table 2). Some newly diagnosed patients were included in a study of ibrutinib in combination with temozolomide, etoposide, liposomal doxorubicin, rituximab, and intrathecal cytarabine (DA-TEDDI-R) but the regimen was associated with high rates of toxicity, specifically aspergillosis in 39% of treated patients [40]. The same combination is now being used with prophylactic anti-fungal agents (NCT02203526). In the upfront setting, ibrutinib is also being studied in combination with MTX, vincristine, procarbazine, rituximab (NCT02315326, NCT04446962), and is being studied as maintenance therapy following response to induction therapy (NCT02623010).

It is unclear whether the next generation of BTK inhibitors such as tirabrutinib and acalabrutinib will offer any advantage over ibrutinib. Tirabrutinib was recently studied in a phase I/II dose escalation trial in Japan for treatment of relapsed/refractory PCNSL. Overall response rate (ORR) was 64% though PFS was only 2.9 months. Tirabrutinib is highly selective for BTK, theoretically reducing toxicity. Nevertheless, nearly half the patients (47.7%) experienced a grade 3 or greater adverse event including three cases of grade 3 skin reaction (2, erythema multiforme) and one case of a grade 5 interstitial lung disease and concurrent *Pneumocystis jirovecii* (PJP) in a patient not treated with PJP prophylaxis [61]. A phase II study in the United States is anticipated (NCT04947319). Acalabrutinib, another second generation BTK inhibitor, is currently being studied in patients with relapsed/refractory primary and secondary CNS lymphoma (NCT04548648, NCT04462328).

### 3.2. PI3K/mTOR Pathway

PI3K is a family of kinases that function as second messengers in multiple signal transduction pathways. Mammalian target of rapamycin (mTOR) is a ubiquitously expressed member of the PI3K family of proteins and a potential therapeutic target. The PI3K/AKT/mTOR pathway is highly conserved regulating cell growth and proliferation [64]. It functions via influence on BTK resulting in activation of NFκB via the BCR pathway but also leads to the activation of independent signaling pathways [41,42]. Inhibition of mTOR has demonstrated modest activity in the treatment of mantle cell lymphoma and systemic DLBCL [65,66].

Temsirolimus, an mTOR inhibitor, was the first targeted agent studied in the treatment of PCNSL. A phase 2 study of relapsed/refractory PCNSL patients yielded a response rate of 54%, notably higher than that observed with systemic lymphoma, but with a PFS of only 2.1 months. Importantly, CSF pharmacokinetics in fourteen samples failed to confirm presence of temsirolimus in all but one specimen which contained a marginal concentration of drug [54]. This was in contrast to a glioma study which demonstrated presence of intratumoral temsirolimus with tissue/blood concentration ratios ranging from 0.69–3.37 [67]. The mismatch between observed response and duration of control may speak to the importance of selecting a therapeutic agent that will treat both the intraparenchymal and leptomeningeal compartments or be a function of early development of resistance mechanisms.

A study of buparlisib, a pan-PI3K inhibitor resulted in even lower response rates (25%) [68]. Again, while pharmacokinetic data from a surgical glioma study demonstrate intratumoral concentrations on par with those in plasma [69], CSF concentrations were subtherapeutic in the CNS lymphoma population [68]. Further complicating the picture is evidence indicating incomplete blockade of the PI3K/AKT/mTOR pathway, even when intratumoral concentrations are achieved [69].

Current studies are underway with additional agents targeting this pathway. PQR309, a dual PI3K/mTOR inhibitor, has shown promise in the preclinical setting. Paxalisib is a PI3K/mTOR inhibitor with CNS penetrance. Each are being studied as monotherapy for patients with relapsed/refractory PCNSL (NCT02669511, NCT04906096). Copanlisib, another PI3K inhibitor, is being used in combination with ibrutinib (NCT03581942) in order to address increased activation of the PI3K/AKT/mTOR pathway observed in *CD79B* mutant lymphomas. Preclinical data suggest synergistic cell death with dual PI3K pathway inhibition and ibrutinib [55].

### 3.3. Immunomedulatory Drugs

Lenalidomide and pomalidomide are second and third generation immunomodulatory drugs (IMiDs) with the potential for direct and indirect antineoplastic effects. IMiDs suppress IRF4 which interfaces with NFκB, as well as MYC, frequently upregulated in PCNSL [8]. They also block the PI3K/AKT pathway, resulting in anti-angiogenic effects [70], and appear to impact the immune microenvironment by modulating tumor-associated macrophages [71].

Lenalidomide has been studied as monotherapy for treatment of recurrent/relapsed PCNSL and SCNSL. Response was seen in 9 of 14 patients (64%) including within the leptomeningeal and ocular compartments. CSF analysis suggested dose-dependent increases in lenalidomide concentration with a CSF/plasma partition coefficient of >20% following the 15 and 20 mg dose levels [56]. A phase 2 study of lenalidomide in combination with systemic rituximab for relapsed/refractory PCNSL yielded an ORR 35.6% with median PFS and OS 7.8 and 17.7 months with a follow up of 19.2 months [58]. The combination was well tolerated and is now being studied in conjunction with ibrutinib (NCT03703167) for treatment of relapsed/refractory PCNSL. A retrospective study of rituximab/lenalidomide/ibrutinib demonstrated response in 8 of 14 heavily pre-treated patients [72]. Multiple combinations using lenalidomide are being studied for both newly diagnosed and relapsed disease (Table 2).

Another potential role for lenalidomide is use as a maintenance agent. In a retrospective study, low doses of 5–10 mg daily appeared to potentiate response to salvage therapy, resulting in longer PFS following salvage therapy than with initial treatment [56]. A small prospective cohort of lenalidomide maintenance following induction therapy with lenalidomide and rituximab induction did not yield as positive results [58]. The role of lenalidomide maintenance following induction treatment for newly diagnosed disease is currently under investigation (NCT04120350, NCT03495960, NCT04627753).

Pomalidomide is a third-generation agent that was studied in combination with dexamethasone in a phase I study of relapsed/refractory PCNSL and primary VRL patients [57]. ORR was 48% with a PFS of 5.3 months in all-comers and 9 months in responders. Notably, one patient had pseudoprogression after 4 cycles of treatment. CSF analysis was performed in one patient; pomalidomide was detected with a CSF-to-plasma ration of 19 and 17% [57], consistent with pre-clinical data [71]. Pomalidomide is now being studied in combination with immunotherapy (NCT03798314).

IMiDs seem to be fairly well tolerated with toxicities most commonly consisting of marrow suppression, infection, and fatigue.

## 4. Targeting the Immune System

Increasingly, evidence suggests immune evasion and immune response modulation play a role in PCNSL pathogenesis and *PD-L1* upregulation has been well-described [52]. Two small retrospective studies have reported encouraging outcomes. Nayak et al., treated five patients (four PCNSL, one isolated SCNSL from testicular primary) with the anti-PD-1 agent nivolumab. All five had objective radiographic responses with four patients achieving a CR. PFS appeared promising at >13 months in all patients, and all were alive at a median follow up of 17 months [73]. The study was of course limited by its retrospective nature and several patients received either concurrent therapy (rituximab) or had initiated nivolumab immediately following brain radiation. Still, it lent support for further investigation into use of immunotherapy. A second, more recent retrospective study reported six patients with PCNSL (3) and isolated SCNSL (3) treated with anti-PD-1 therapy, pembrolizumab (5) or nivolumab (1). Ambady et al., achieved CR in three of six patients and reported progressive disease in the remaining. Interestingly, one patient who achieved an initial CR progressed after therapy was discontinued but was able to re-attain a CR upon re-initiation of immunotherapy [74]. PD-1 blockade tends to be well-tolerated and has the potential to offer a viable alternative treatment strategy to patients who are elderly or frail. Prospective studies are ongoing exploring its use as monotherapy (NCT02857426) and in conjunction with other agents such as ibrutinib (NCT03770416, NCT04421560), lenalidomide (NCT04609046), or pomalidomide (NCT03798314). PD-1 blockade is also being explored as a potential maintenance or consolidation strategy (NCT04401774, NCT04022980).

Targeting tumors with chimeric antigen receptor T (CAR-T) cells is a novel strategy that utilizes a patients’ own genetically engineered T cells to identify and bind a tumor-specific target antigen. CD19-targeted CAR-T cells have been studied in systemic DLBCL with encouraging results [75]. Initially patients with CNS disease were excluded from studies out of concern for neurotoxicity and the potential for limited efficacy at immunoprivileged sites. However, CAR-T cells have been identified in the CSF [75] and an index patient with SCNSL and concurrent systemic disease demonstrated a CR in the brain following treatment with CD-19 directed CAR-T cell therapy [76]. More recently, a retrospective report of patients with SCNSL treated with off-label tisagenlecleucel, another CD19-directed CAR-T, yielded responses in four of eight patients (two CR, two partial response at 28 days) [77]. Notably T-cell expansion was evident even in patients with isolated CNS disease. The treatment was tolerated well with no reports of greater than grade 1 neurotoxicity [77]. Preliminary data from an ongoing clinical trial enrolling patients with PCNSL reported high rates of toxicity with all patients developing at least grade 1 cytokine release syndrome and neurotoxicity, though all toxicities were reversible [78]. At initial disease response, three of five patients had achieved CR while the remaining 2 appeared to have stable disease. Additional prospective studies of CD19 CAR-T agents tisagenlecleucel (NCT04134117) and axicabtagene ciloleucel (NCT04608487) are underway in patients with CNS lymphoma, with results eagerly awaited. Newer generations of CAR-T cells are in development and may allow for modulation of the tumor microenvironment simultaneous with direct tumor killing. This newer generation of agents known as T-cells redirected for antigen-unrestricted cytokine-initiated killing (TRUCKs) express an additional transgenic inducible-cytokine to be released upon tumor-antigen binding, inducing a pro-inflammatory response and potentially mitigating the immunosuppressive lymphoma microenvironment [79].

Bi-specific T-cell engagers (BiTEs) are engineered bi-specific monoclonal antibodies with two single-chain variable domains of different antibodies. One domain targets the CD3 receptor on T cells while the other targets a tumor-specific antigen. BiTEs form a link between T cells and tumor, triggering cellular death via target cell lysis in the absence of regular major histocompatibility complex (MHC) class I/peptide antigen recognition [80]. Blinatumomab, a CD19/CD3-BiTE has been approved for use in the treatment of B-cell precursor acute lymphoblastic leukemia with minimal residual disease. It and a variety of CD20/CD3-BiTEs are undergoing investigation for treatment of systemic DLBCL [81]. At this time, studies are not enrolling patients with CNS disease due to concerns for neurotoxicity; however, this may be a treatment strategy in the future.

## 5. Other Targets

Other potential therapeutic targets are being explored in PCSNL. Loss of CDKN2A is frequently observed [52] and may be targeted by cyclin dependent kinase inhibitors. A small prospective study of abemaciclib in CNS lymphoma is ongoing (NCT03220646). Venetoclax, a targeted agent against BCL-2, appears to penetrate the BBB—though at lower concentrations—and may have some efficacy in CNS lymphoproliferative disease [82,83]. A prospective study of venetoclax with obinutuzumab, an anti-CD20 monoclonal antibody, was halted due to low enrollment (NCT04073147).

Selinexor, an inhibitor of exportin 1, blocks nuclear export, leading to accumulation of tumor suppressor proteins in the nucleus and resultant cell death. It is currently approved for the treatment of refractory multiple myeloma, relapsed systemic diffuse large B-cell lymphoma, and is planned to be studied for treatment of PCNSL. Pre-clinical data suggest selinexor may have synergy with ibrutinib, potentially paving the way for future studies [84].

## 6. Challenges to Drug Development and Delivery

Development of new targeted treatments has been difficult. One challenge is that PCNSL is a rare disease, limiting the ability to perform statistically significant head-to-head comparisons of treatment strategies. Prior to large-scale clinical studies however, it is important to achieve adequate understanding of drug pharmacokinetics in the CNS. Many targeted drugs such as proteasome inhibitors are too large to penetrate the BBB. Increasingly, it is being recognized that drug concentrations need to be explored in both the leptomeningeal compartment and intraparenchymal tumor tissue as one appears to be a poor surrogate for the other. Differences in concentration may be a result of frequent breakdown of the BBB in intraparenchymal disease.

Penetration of the BBB remains a challenge in the treatment of CNS malignancies, including PCNSL. One potential strategy to enhance drug delivery is disruption of the BBB, which can potentially be achieved with drugs, ultrasound, or osmotic disruption. One multi-center study of BBB disruption (BBBD) using mannitol followed by intra-arterial (IA) MTX yielded an ORR of 81.9% (CRR 57.8%) with an OS of 3.1 years [85]. This compared favorably to historical controls, particularly considering that approximately half the patients enrolled did not undergo consolidation treatment. Another strategy for BBBD include delivery of low doses of tumor necrosis factor-alpha (TNF) to the vasculature. This has been followed by delivery of systemic lymphoma regimens with otherwise poor CNS penetration (rituximab/cyclophosphamide/doxorubicin/vincristine/prednisone or R-CHOP) with good response rates [86].

Development of drug resistance is also a complicating factor, particularly for molecular strategies targeting only a single pathway. Combination studies are one potential strategy to reduce resistance. For example, while ibrutinib is associated with a short PFS when used as monotherapy, response appears more durable when it is used in combination. As a result, ibrutinib is now under investigation as part of a number of potential treatment regimens (Table 2). These studies are ongoing, and it remains to be seen whether this strategy will improve efficacy and long-term control in PCNSL.

## 7. Future Directions

The efficacy of MTX has meant that the investigation of most of these novel treatment strategies has been in the relapsed/refractory setting. Only recently are studies starting to incorporate the use of some of these newer agents into upfront treatment, and largely in combination with MTX. It remains to be seen whether any of these agents will obviate the need for MTX and for the most part, this is not being studied except in patients who are considered ineligible for MTX-based therapy. While MTX is effective, it necessitates frequent hospitalizations, leading to time away from work and family. Additionally, it confers risk of MTX-related toxicity, as well as complications associated with inpatient admission such as delirium, urinary tract infections, and thromboembolic events. Many of the novel therapies are oral and most can be administered in the outpatient setting. If they prove to be as effective as MTX, this may lead to a new treatment paradigm for PCNSL.

As we continue to develop novel strategies for this disease, it will become increasingly important to develop minimally invasive biomarkers. Traditionally, patients are monitored for recurrence with routine MRIs and possibly CSF sampling and ocular exams, depending on their presentation. Monitoring of biomarkers such as interleukin-10 (IL-10) may help monitor treatment response and allow for early detection of relapse [56,87]. Detection of circulating tumor DNA (ctDNA) may serve a similar role while allowing for detection and confirmation of genetic arrangements. While this technology has been unsuccessful in the serum of patients with PCNSL [88] in CSF, ctDNA has been used to detect molecular alterations [59,89]. Studies are ongoing to determine whether detection of ctDNA in the CSF is of prognostic import and can be used to monitor treatment response (NCT04401774). Monitoring of ctDNA in the CSF may also allow for monitoring of the presence of targetable mutations.

## 8. Conclusions

Advances in our understanding of the molecular drivers of PCNSL have led to the development of novel drug strategies. We must ensure these drugs penetrate the CNS, create responses, and that these responses are durable. Combination therapy may be one way to avoid early resistance. Harnessing of the immune system is another strategy. Further genetic characterization and monitoring will be crucial in furthering our understanding and predicting response.

## Figures and Tables

**Table 1:** Recent prospective trials of novel agents.

Author	Year	Agent(s)	Phase	Evaluable Patients	Disease Status	Median Age, y	ORR (PR + CR)	mPFS, mo	mOS, mo
Korfel [54]	2016	Temsirolimus	2	37	R/R	70	20/37 (54%)	2.1	3.7
Grommes [55]	2017	Ibrutinib	1	20 (13 PCNSL)	R/R	69	10/13 (77%)	4.6	15
Lionakis [40]	2017	TMZ, etoposide, liposomal doxorubicin, dexamethasone, rituximab, ibrutinib	1b	18	R/R, new	66	15/18 (83%)	15.3 in R/R	NR
Rubenstein [56]	2018	Lenalidomide + rituximab; lenalidomide maintenance	1	14 (7 PCNSL)	R/R	66	6/7 (86%)	6	NS
Tun [57]	2018	Pomalidomide + dexamethasone	1	25 (23 PCNSL)	R/R	NS, >60	11/23 (48%)	5.3	NS
Ghesquieres [58]	2019	Lenalidomide + rituximab	2	45 (34 PCNSL)	R/R	69	22/34 (65%)	3.9	NS
Grommes [59]	2019	Ibrutinib + M(3.5) + rituximab	1b	15 (9 PCNSL)	R/R	62	8/9 (89%)	NR	NR
Soussain [60]	2019	Ibrutinib	2	44	R/R	70	26/44 (59%)	4.8	19.2
Narita [61]	2021	Tirabrutinib	1/2	44	R/R	60	28/44 (64%)	2.9	NR

CR: complete response; M: methotrexate; mOS: median overall survival; mo: months mPFS: median progression-free survival; NR: not reached; NS: not specified; ORR: overall response rate; PCNSL: primary central nervous system lymphoma; PR: partial response; R/R: relapsed/refractory; TMZ: temozolomide; y: years.

**Table 2:** Ongoing trials of novel agents.

Agents	Clinicaltrails.Gov ID	Trial Start	Phase	Target Accrual	Eligible Age	Country
**Upfront Induction**						
Rituximab, MTX, lenalidomide, nivolumab	NCT04609046	2020	1	27	18+	USA
Rituximab, MTX, procarbazine, vincristine; and lenalidomide or ibrutinib	NCT04446962	2020	1b/2	128	18 to 60	France
Rituximab, MTX ± lenalidomide	NCT04481815	2020	2	240	18 to 75	China
Rituximab, lenalidomide, MTX, and TMZ	NCT04737889	2021	2	30	18 to 70	China
Rituximab, MTX, procarbazine, vincristine, and ibrutinib	NCT02315326	2021	2	30	18+	USA
**Upfront Maintenance**						
Nivolumab maintenance	NCT04022980	2019	1b	20	65+	USA
MTX, rituximab, lenalidomide, with lenalidomide maintenance	NCT04120350	2019	1b/2	47	18 to 75	China
Rituximab, MTX, with ibrutinib maintenance	NCT02623010	2016	2	30	60 to 85	Israel
MTX or TMZ-based therapy with procarbazine or lenalidomide maintenance	NCT03495960	2019	2	208	70+	Italy
Lenalidomide/rituximab maintenance	NCT04627753	2020	2	30	19+	Korea
Nivolumab maintenance	NCT04401774	2020	2	25	18+	USA
**Relapsed/Refractory Disease**						
TMZ, etoposide, liposomal doxorubicin, dexamethasone, ibrutinib, rituximab, IT-cytarabine	NCT02203526	2014	1	93	18+	USA
Tisagenlecleucel	NCT04134117	2019	1	6	18+	USA
Acalabrutinib and durvalumab	NCT04462328	2020	1	21	18+	USA
Fludarabine, cyclophosphamide, axicabtagene ciloleucel	NCT04608487	2020	1	18	18+	USA
Ibrutinib with rituximab and lenalidomide	NCT03703167	2019	1b	40	18+	USA
Copanlisib with ibrutinib	NCT03581942	2018	1b/2	45	18+	USA
Pembrolizumab, ibrutinib, and rituximab	NCT04421560	2020	1b/2	37	18+	USA
PQR309	NCT02669511	2015	2	21	18+	Germany
Nivolumab	NCT02857426	2016	2	47	18+	USA
Abemaciclib	NCT03220646	2017	2	10	18+	USA
Ibrutinib, rituximab, ifosfamide and etoposide, with ibrutinib maintenance	NCT04066920	2019	2	30	20 to 79	Korea
Nivolumab and ibrutinib	NCT03770416	2019	2	40	18+	USA
Nivolumab and pomalidomide	NCT03798314	2019	1	3	18+	USA
Acalabrutinib	NCT04548648	2020	2	32	18+	USA
Ibrutinib versus lenalidomide, with MTX, rituximab, etoposide	NCT04129710	2020	2	120	18 to 75	China
Orelabrutinib	NCT04438044	2020	2	39	18 to 75	China
Paxalisib	NCT04906096	2021	2	25	18+	USA
Tirabrutinib	NCT04947319	2021	2	44	18+	USA

IT: intrathecal; MTX: methotrexate; TMZ: temozolomide.
